# Romantic relationship dissolutions are significantly associated with posttraumatic stress symptoms as compared to a DSM-5 Criterion A event: a case-case–control comparison

**DOI:** 10.1080/20008066.2023.2238585

**Published:** 2023-08-01

**Authors:** Alberta Susanna Johanna Van der Watt, Martin Kidd, Annerine Roos, Elmien Lesch, Soraya Seedat

**Affiliations:** a Department of Psychiatry, Faculty of Medicine and Health Sciences, Stellenbosch University, Tygerberg, South Africa; b Centre for Statistical Consultation, Stellenbosch University, Stellenbosch, South Africa; c Department of Psychiatry and Neuroscience Institute, University of Cape Town, Cape Town, South Africa; d Department of Psychology, Stellenbosch University, Stellenbosch, South Africa; e SAMRC Genomics of Brain Disorders Research Unit, Department of Psychiatry, Faculty of Medicine and Health Sciences, Stellenbosch University, Tygerberg, South Africa

**Keywords:** Attachment, breakup, emerging adults, non-marital, posttraumatic stress disorder, South Africa, university students, Apego, ruptura, adultos emergentes, pareja, trastorno de estrés postraumático, Sudáfrica, estudiantes universitarios, 依恋, 分手, 新成年人, 非婚姻, 创伤后应激障碍, 南非, 大学生

## Abstract

**Background:** Non-marital romantic relationship dissolutions (RRDs) are common among emerging adult students (EAS) and may result in severe distress and suicidality. However, studies on RRDs in youth are limited to mental health sequelae of depression and prolonged grief. Little is known about the association between RRDs and posttraumatic stress symptoms (PTSS), and how this compares to posttraumatic stress symptoms following a traumatogenic event.

**Objective:** We aimed to determine the association between RRDs and PTSS in an EAS sample; and how this compared to the association between posttraumatic stress symptoms and a Diagnostic and Statistical Manual 5th edition (DSM-5) traumatic event.

**Method:** University students (*N *= 2,022; female = 71.1%; 18–25 years) completed a demographic and relationship questionnaire, the Life Events Checklist, the Adverse Childhood Experiences questionnaire, and the Posttraumatic Stress Checklist (PCL). We compared EAS with an RRD (*n *= 886) or a DSM-5 criterion A traumatic event (*n *= 592) against a control group (*n *= 544) exposed to a non-traumatic stressful life event. Utilising ANOVAs and Pearson’s correlations we determined demographic and clinical variables associated with PTSS. ANCOVA and stepwise hierarchical regression analyses were used to determine between-group differences in PTSS.

**Results:** Total trauma exposure and adverse childhood experiences, sex, monthly income, sexual orientation, and attachment style were significantly associated with PTSS. The RRD group had significantly higher PCL scores compared to the DSM and control groups. The mean PCL scores for both the RRD and DSM groups were above the cut-off score of 33, consistent with a probable posttraumatic stress disorder diagnosis. Significantly more RRD participants (72.9%) scored above the cut-off score of 33 than DSM-5 Trauma Group participants (55.4%).

**Conclusion:** An RRD is a potentially traumatic event and is significantly associated with PTSS, similar to a posttraumatic stress disorder diagnosis.

## HIGHLIGHTS

- Students may experience non-marital romantic relationship breakups as very painful, with various negative psychological, physical, and academic consequences. However, their painful experiences are often trivialised as something to ‘just get over’ and not acknowledged as potentially traumatic. Yet, using an attachment theory framework, breakups can be reconceptualised as subjectively traumatic.
- We explored the differences in posttraumatic stress symptoms of students who experienced a breakup, a traumatic event (as defined in psychiatry), and other non-traumatic life events. Students who experienced a breakup reported significantly highly posttraumatic stress symptoms.
- Acknowledging students’ pain following a self-defined traumatic breakup can increase help-seeking behaviour and reduce the risk of intergenerational attachment trauma. Our findings warrant further research of breakups as potentially traumatogenic events.

## Abstract (es)

**Antecedentes:** Las disoluciones de relaciones románticas no matrimoniales (RRD en su sigla en inglés) son comunes entre los estudiantes adultos emergentes (EAS en su sigla en inglés) y pueden provocar angustia grave y tendencias suicidas. Sin embargo, los estudios sobre RRD en jóvenes se limitan a las secuelas de salud mental de la depresión y el duelo prolongado. Se sabe poco sobre la asociación entre los RRD y los síntomas de estrés postraumático (PTSS en su sigla en inglés), y cómo se compara esto con los síntomas de estrés postraumático después de un evento traumatogénico.

**Objetivo:** Nuestro objetivo fue determinar la asociación entre RRD y PTSS en una muestra de EAS; así como también cómo se compara esto con la asociación entre los síntomas de estrés postraumático y un evento traumático de la quinta edición del Manual Diagnóstico y Estadístico (DSM-5).

**Método:** Estudiantes universitarios (*N *= 2,022; mujeres = 71.1%; 18–25 años) completaron un cuestionario demográfico y de relaciones, la Lista de Verificación de Eventos Vitales, el cuestionario de Experiencias Adversas en la Infancia y la Lista de Verificación de Estrés Postraumático (PCL). Comparamos EAS con un RRD (*n* = 886) o un evento traumático del criterio A del DSM-5 (*n* = 592) contra un grupo de control (*n* = 544) expuesto a eventos de vida estresantes no traumáticos. Utilizando ANOVA y correlaciones de Pearson, determinamos variables demográficas y clínicas asociadas con PTSS. Se utilizó ANCOVA y análisis de regresión jerárquica paso a paso para determinar las diferencias de PTSS entre grupos.

**Resultados:** La exposición total al trauma y la infancia adversa experimentada, el sexo, los ingresos mensuales, la orientación sexual y el estilo de apego se asociaron significativamente con PTSS. El grupo RRD tuvo puntuaciones PCL significativamente más altas en comparación con los grupos DSM y control. Las puntuaciones PCL medias para los grupos RRD y DSM estuvieron por encima de la puntuación límite de 33, lo que concuerda con un diagnóstico probable de trastorno de estrés postraumático. Significativamente más participantes de RRD (72.9%) puntuaron por encima de 33 puntos que los participantes del Grupo de Trauma del DSM-5 (55.4%).

**Conclusión:** Un RRD es un evento potencialmente traumático y está significativamente asociado con PTSS, similar a un diagnóstico de trastorno de estrés postraumático.

## Abstract (zh)

**背景：**非婚姻恋爱关系破裂（RRD）在新成年学生（EAS）中很常见，可能导致严重的痛苦和自杀倾向。 然而，对青少年 RRD 的研究仅限于抑郁和延长哀伤的心理健康后遗症。人们对 RRD 与创伤后应激症状 (PTSS) 之间的关联以及其与创伤性事件后的创伤后应激症状的比较知之甚少。

**目的：**我们旨在确定 EAS 样本中 RRD 与 PTSS 之间的关联； 以及这个关联与创伤后应激症状与一项诊断和统计手册第五版 (DSM-5) 创伤事件之间的关联进行比较。

**方法：**大学生（*N* = 2,022；女性 = 71.1%；18–25 岁）完成了人口统计和关系问卷、生活事件清单、不良童年经历问卷和创伤后压力清单 (PCL)。 我们将有RRD的 EAS组 (*n* = 886) 或有DSM-5 标准 A 创伤性事件的EAS组 (n = 592) 与暴露于非创伤性压力生活事件的对照组 (*n* = 544) 进行比较。利用方差分析和皮尔逊相关性，我们确定了与 PTSS 相关的人口统计和临床变量。使用协方差分析和逐步分层回归分析来确定PTSS的组间差异。

**结果：**总创伤暴露和不良童年经历、性别、月收入、性取向和依恋方式与 PTSS 显著相关。与 DSM 组和对照组相比，RRD 组的 PCL 评分显著更高。 RRD 组和 DSM 组的平均 PCL 分数均高于 33 分的临界分数与可能的创伤后应激障碍诊断一致。 得分高于 33 分的 RRD 参与者 (72.9%) 显著多于 DSM-5 创伤组参与者 (55.4%)。

**结论：**RRD 是一种潜在的创伤事件，与 PTSS 显著相关，类似于创伤后应激障碍的诊断。

## 1. Background

A diagnosis of posttraumatic stress disorder (PTSD) can be made following exposure to a traumatic event according to criterion A of the Diagnostic and Statistical Manual 5th edition (DSM-5) (American Psychiatric Association, 2013). Specifically, a traumatic event should be life-threatening and is defined as: ‘… exposure to actual or threatened death, serious injury, or sexual violence’ (American Psychiatric Association, 2013). However, there is evidence that significant posttraumatic stress symptoms (PTSS) can emerge following exposure to non-criterion A events, such as oppression (Holmes et al., 2016), loss/death of a pet, unwanted relocation (Lansing et al., 2017), unemployment, homelessness (Alessi et al., 2013), separation from parents/loved ones, or ending a relationship (Alessi et al., 2013; Chung et al., 2003, Chung et al., 2002; Lansing et al., 2017). Indeed, a meta-analysis found a stronger relationship between PTSS and traumatic events than DSM-5 incongruent traumas (‘stressors’), however, the effect size was small (0.18) and there was an overlapping distribution of PTSS in the ‘trauma’ and ‘stressor’ groups (Larsen & Pacella, 2016). Thus, the ‘utility of criterion A, as currently defined [in the DSM-5] as the syndromal gatekeeper for PTSD symptomatology’ (p. 21) is called into question (Zelazny & Simms, 2015).

Romantic relationship dissolutions (RRDs) fall into this category of non-criterion A events that may be associated with significant PTSS. Emerging adults (Arnett, 2000) experiment with romantic partners (Carver et al., 2003; Meier & Allen, 2008) during a challenging, uncertain, and transitional period of life (Simon & Barrett, 2010) when they are at an elevated risk for mental health concerns (Field et al., 2009). Based on Arnett’s (2000) theory, the developmental stage of emerging adulthood is distinct for identity exploration in three main areas: love, work, and worldview. In the process of exploring romantic identities, emerging adults implicitly engage with existential issues such as who they are, what they want from romantic relationships, and who they want as partners in romantic relationships (Arnett, 2000). Therefore, much is at stake for emerging adults in the romantic relationship realm.

RRDs, albeit not homogenous in nature (Belu et al., 2016), may be experienced as severely distressing among university students (Fang et al., 2018; Pérez-Rojas et al., 2017; LeFebvre et al., 2019; Norona et al., 2017). Indeed, research indicates that an RRD is a risk factor for suicidality (see Kazan et al., 2016 for review). However, since the DSM-5 does not recognise an RRD as a traumatogenic event, research on RRDs in adolescents predominantly focus on psychological consequences such as depression and prolonged grief (McConnell et al., 2018; O’Connor & Sussman, 2014; Robinaugh et al., 2015). Still, there is limited research demonstrating a clear link between RRDs and PTSS (Chung et al., 2002; Chung, Farmer, et al., 2002; Chung et al., 2003; Studley & Chung, 2015). Further, a recent systematic review of functional magnetic resonance imaging (fMRI) and romantic rejection indicated overlapping neural activation in regions implicated in PTSD (including the fear-based limbic system) (van der Watt et al., 2021). Yet, these studies on the association between an RRD and PTSS have largely been limited to the United Kingdom (Chung et al., 2002; Chung, Farmer, et al., 2002; Chung et al., 2003) and the United States of America (USA) (Studley & Chung, 2015). Specifically, Chung et al. (2002) reported significant levels of intrusive thoughts and avoidance behaviours, with higher scores for intrusive thoughts on the Impact of Event Scale. Similar research among emerging adult students (EAS) in developing countries is lacking. To fill this gap in research, we use attachment theory – developed by Bowlby (1979) and based on evolutionary principles – as theoretical framework.

### 1.1 Attachment theory as theoretical framework

Based on Bowlby’s theory (1969, 1973, 1980), a child attaches to a caregiving figure to survive. The child’s attachment behavioural system consists of behaviours aimed at physical proximity to a protective figure and the psychological goal of felt security (Bowlby, 1973; Cassidy, 1999; Cassidy, 2000). Thus, from an attachment theory perspective a child may experience separation from a caregiver as threatening to their survival or security and, subsequently, experience high levels of distress.

Hazan and Shaver (1987) extended Bowlby’s theory to adult romantic attachment theory postulating that emotional and behavioural dynamics of adult romantic relationships are directed by the same biological and survival systems (Fraley & Shaver, 2000). Adults seek physical or psychological proximity to a romantic partner (Fraley & Shaver, 2000) to promote survival and safety (Bowlby, 1969). Adults typically feel safer and more secure when their partners are close by, accessible, and responsive. When unwell, threatened, or distressed, they will seek out their partners for comfort, safety, and protection (Hazan & Shaver, 1987) – indeed, research indicates that young adults show a preference to a romantic partner for proximity and as a secure base (Fagundes & Schindler, 2012). It follows that when their attachment figure is not available, they may experience their safety to be threatened. Therefore, from an attachment theory framework, separation from a romantic partner (such as an RRD) may constitute a *life-threatening* event (Van der Watt et al., 2022), and thereby qualify as a life-threatening DSM-5 Criterion A event for PTSD. The reconceptualisation of RRDs as potentially traumatic events (using attachment theory as framework, including the overlap in neurocircuitry) and the importance of focusing on RRD research within a trauma paradigm is discussed in detail in a recently published narrative review (van der Watt et al., 2022).

Attachment theory centres on concepts of secure and insecure (avoidant and anxious-ambivalent) attachment styles and internal working models (IWMs) (Bowlby, 1973; Hazan & Shaver, 1987; Ainsworth et al., 1978; Bartholomew, 1997; Fraley and Shaver (2000). According to attachment theory, attachment styles and IWMs influence emotion regulation in dealing with stressful events (Shaver & Mikulincer, 2007), distress levels following an RRD (Heshmati et al., 2022), and the development of PTSD following a traumatic event (O’Connor & Elklit, 2008; Woodhouse et al., 2015). For example, avoidantly attached individuals may de-activate their attachment-related thoughts and emotions along with negative reactions such as greater self-blame and use of drugs and alcohol to cope following an RRD (Marshall et al., 2013).

However, individuals with anxious-ambivalent attachment styles may be at particular risk of PTSS since they tend to react to RRDs with hyperactivated emotional and physiological distress, drug and alcohol abuse, a lost sense of identity, and preoccupation with the ex-partner (Davis et al., 2003; Marshall, 2012; Saffrey & Ehrenberg, 2007; Sbarra & Emery, 2005; Spielmann et al., 2009). This is consistent with Bowlby’s theory whereby anxious individuals are prone to prolonged protest, despair, and continued attachment to the ex-partner (Marshall et al., 2013). This amplified distress can be attributed to their poor coping strategies, maladaptive ruminations about the ex-partner, dysfunctional reliance on the ex-partner, and the tendency to blame themselves for any negative events that may occur (Davis et al., 2003; Faugundes, 2012; Saffrey & Ehrenberg, 2007; Spielmann et al., 2009). Thus, EAS with an anxious-ambivalent attachment style may be particularly vulnerable to PTSS following a stressful event.

Additionally, IWMs may play a role in the therapeutic context influencing receptiveness to different types of interventions, with insecure IWMs generally being more challenging to address (Wallin, 2007). Since involuntary attachment separation, such as RRDs, may negatively influence the construction of IWMs (Mikulincer & Shaver, 2007) and have implications for future well-being, a better understanding of the impact of RRDs among EAS is important.

### 1.2 Factors associated with PTSS

Certain factors may contribute to PTSS following exposure to an RRD, a DSM-5 criterion A event, or another stressful event and must be considered when conducting research related to PTSS. For example, traumatic and stressful events are known to be positively associated with PTSS (American Psychiatric Association, 2013; Lansing et al., 2017, 2016). Specifically, research indicates that cumulative adversity (Lansing et al., 2017), including childhood adversity, increases the risk of psychopathology and PTSD in response to new stressors (Duke et al., 2010; Lansing et al., 2016; Noble et al., 2015). Further, as mentioned before, attachment style can influence emotion regulation, dealing with stressful events (Shaver & Mikulincer, 2007), and the development of PTSD (O’Connor & Elklit, 2008; Woodhouse et al., 2015).

In terms of socio-demographic factors, research points to sex differences in PTSS following traumatic and stressful events (Irish et al., 2011), including RRDs (Chung et al., 2002). Specifically, women are more likely to develop PTSS following exposure to traumatic events (Christiansen & Berke, 2020; Christiansen & Elklit, 2011; Shansky, 2015). Further, women report higher levels of chronic strain, rumination (Nolen-Hoeksema et al., 1999), distress, depression (Kendler et al., 2003), and depression-like symptoms (Verhallen et al., 2019) following an RRD. However, one study found similarly intense emotional responses in men following an RRD (Morris et al., 2015), while another indicated that men also struggle to recover from an RRD (Barber & Cooper, 2014). Additionally, while being in a relationship may serve as a protective factor in situations of extreme stress (Israel-Cohen & Kaplan, 2016) and play a role in the development of PTSS (Weisenhorn et al., 2017), it appears to be more of a protective factor for men than for women (Israel-Cohen & Kaplan, 2016). This highlights the importance of also considering current relationship status when conducting research related to PTSS.

Sexual orientation may also influence the reaction to stressful events (Roberts et al., 2010) such as RRDs (Lannutti & Cameron, 2002), especially when considering minority stress theory (Alessi et al., 2013). Specifically, minority stress theory postulates that socially disadvantaged populations (such as sexual and racial/ethnic minorities) have excess exposure to stress (such as homophobia). Therefore, persons who identify as lesbian, gay, bisexual, transsexual, queer, and intersexual plus (LGBTQI+) may be at an increased risk of mental health problems (Gnan et al., 2019; Pachankis et al., 2020) following traumatic and/or stressful events.

Lastly, time since trauma exposure may play a role in the development of PTSS following a traumatic (Weems & Carrion, 2007) or stressful event (Field et al., 2009). Specifically, in terms of an RRD, a shorter time since the dissolution has been associated with greater distress (Field et al., 2009; Moller et al., 2003). All these factors were considered in our study and incorporated into the hypotheses.

### 1.3 Aims and hypotheses

The above literature underscores the importance of determining the association between RRDs and PTSS in an EAS sample. How this compares to the association between PTSS and a DSM-5 traumatic event is also not known. Further, to identify at-risk populations, it is crucial to understand the factors (such as trauma-related exposure, attachment style, and sociodemographic variables) that may be associated with increased levels of PTSS. Thus, this study sought to answer the following questions:

1. Are trauma-related exposures, attachment style, and socio-demographic factors (including sex, sexual orientation, and current relationship status) associated with PTSS?^1^
2. Is there a difference in PTSS following an RRD or a DSM criterion A trauma event, compared to a control group exposed to stressful (non-traumatic) life events?

We hypothesised that:

1. Trauma-related exposures, an anxious-ambivalent attachment style, being female, having a non-heterosexual orientation, and being single would show a positive correlation with PTSS scores.
2. PTSS scores on the Posttraumatic Stress Checklist for DSM-5 (PCL-5) would be similar in EAS with an RRD compared with EAS who have experienced a DSM-5 criterion A trauma.

## 2. Methodology

Data were drawn from Phase 1 of a larger study with the overarching aim to explore the difference in PTSS, neural circuitry, and the experience of traumatic stress following an RRD as compared to a DSM-5 criterion A event in a South African university sample. The data reported here were collected between 1 August 2019 and 20 March 2020.

### 2.1 Sample

We used purposive sampling. A mass email invitation was sent to all registered Stellenbosch University students aged 18–25 years to complete an online survey. Of the 27 256 students who received the invitation 2 152 (7.9%) responded. Eighteen responses were excluded due to participants falling outside the age range; and 112 responses were excluded due to incomplete data (e.g. not answering a full scale). This resulted in a total sample of 2 022 participants.

As indicated in Table 1, the sample mainly consisted of female (71.1%), white (60.6%), currently single (56.6%), and heterosexual (79.9%) EAS. The sample had a mean age of 20.55 years (age range = 18–25 years; SD = 1.89 years). Most participants (*n *= 907; 44.9%) identified with an avoidant attachment style, followed by secure (*n *= 759; 37.5%), and anxious-ambivalent (*n *= 356; 17.6).

**Table 1.** Socio-demographic information for the full sample.

Variable	*n*(%)	Variable	*n*(%)
Sex Male Female Intersexual	581(28.7) 1 438(71.1) 3(0.1)	Religious orientation^a^ I am not religious Christian Other^b^	541(26.8) 1 298(64.2) 116(5.7)
Race^c^ African/black Mixed race/coloured^d^ Indian White Other Prefer not to say	310(15.3) 355(17.6) 62(3.1) 1 226(60.6) 22(1.1) 44(2.2)	Monthly income of main breadwinner^e^ Do not know R 0–R 11 352 (0 USD-750.70 USD) R 11 353–R 30 164 (750.76 USD–1 994.72 USD) R 30 165–R 68 528 and above (1 994.79 USD–4 531.70 USD and above)	435(21.5) 309(15.3) 486(24.0) 780(38.6)
Sexual orientation^f^ Heterosexual Other^g^	1 616(79.9) 389(19.7)	Current relationship status Single Not single^h^	1 145(56.6) 877(43.4)

Note: USD = United States Dollar (USD equivalents were calculated on the exchange rate as on 1 March 2021).

^a^ Sixty-seven (3.3%) participants did not answer this question.

^b^ Including Jewish, Mulsim, Hindu, and other.

^c^ Three participants (0.1%) did not answer this question.

^d^ The term ‘coloured’ appeared in the late nineteenth century. While the referral to racial groups within South African scholarship may seem controversial, the term is still used to denote or identify oneself as belonging to a particular racial group. The term is not made use of to support the Apartheid ideology, but rather to recognise a history of economic and political differences between racial groups within South Africa (Furphy, 2012).

^e^ Twelve (0.6%) participants did not answer this question.

^f^ Eight (0.4%) participants did not answer this question.

^g^ Including homosexual, bi-sexual, pansexual, A-sexual, and other.

^h^ Including dating one person, in an exclusive/committed relationship, in multiple committed relationships (polyamory), and engaged.

### 2.2 Procedure

After approval by the Health Research Ethics Committee at Stellenbosch University (S19/01/021), the email invitation was sent to students that included information regarding the study and a link to the online survey. The landing page consisted of a detailed informed consent form highlighting the voluntary and confidential nature of the study. Participants provided digital informed consent. Participants were not compensated for their time but were invited to enter a lucky draw with eight participants winning a voucher of R500 (approximately 28.31 USD at the time) each.

### 2.3 Measures

Participants completed a demographic and relationship questionnaire consisting of four sections (i) basic socio-demographic information; (ii) psychiatric and medical information; (iii) current relationship information; and (iv) previous relationship information. Attachment style was determined by means of a widely used single item valid measure developed by Hazan and Shaver (1987) consisting of three statements for secure, anxious-ambivalent, and avoidant romantic attachment styles respectively (Hazan & Shaver, 1987)^2^ Specifically, participants indicated the statement that best described them:

Secure: ‘I find it relatively easy to get close to others and am comfortable depending on them and having them depend on me. I don’t worry about being abandoned or about someone getting too close to me’;

Avoidant: ‘I am somewhat uncomfortable being close to others; I find it difficult to trust them completely, difficult to allow myself to depend on them. I am nervous when anyone gets too close, and often, others want me to be more intimate than I feel comfortable being’; and

Anxious-ambivalent: ‘I find that others are reluctant to get as close as I would like. I often worry that my partner doesn’t really love me or won’t want to stay with me. I want to get very close to my partner, and this sometimes scares people away’ (Fraley & Shaver, 2000).

Additionally, participants completed the Life Events Checklist (LEC) (Gray et al., 2004), the Adverse Childhood Experiences Questionnaire (ACE) (WHO, 2011), and the Posttraumatic Stress Checklist for DSM-5 (PCL-5) (Weathers et al., 2013).

#### 2.3.1 Life Events Checklist

The LEC is a reliable measure of exposure to potentially traumatic events (PTEs) developed at the National Center for PTSD to facilitate the diagnosis of PTSD (Gray et al., 2004). The LEC inquires about multiple types of exposure to PTEs. Using a nominal scale (1 = happened to me, 2 = witnessed it, 3 = learned about it happening to someone close, 4 = part of my job, 5 = not sure/not applicable), participants indicated their experience of PTEs (Gray et al., 2004). Responses were coded as 1 = single exposure (happened to me OR witnessed it) and 2 = double exposure (happened to me AND witnessed it). Items indicating learned about it, part of my job, and not sure/not applicable; were coded as 0 = no exposure. Total LEC scores were determined by summing the first 16 items (thereby excluding ‘other stressful events’, since it is not clear whether these meet criterion A requirements). As part of the LEC, participants indicated their self-defined *most traumatic event* in an open-ended question. In the present sample, the Cronbach’s alpha coefficient for the LEC was 0.750.

#### 2.3.2 Adverse Childhood Experiences questionnaire

The ACE was developed by the World Health Organization (WHO, 2011) and measures types of abuse, neglect, violence between parents/caregivers, and other serious sources of stress that children may suffer early in life. Example items include: ‘Did your parents/guardians understand your problems and worries?’; ‘Did you see or hear a parent or household member in your home being yelled at, screamed at, sworn at, insulted, or humiliated?’; and ‘Were you beaten up by soldiers, police, militia, or gangs?’. The ACE has demonstrated good test-retest reliability with *α *= 0.90 (Ho et al., 2019). Binary scoring (0 = no exposure, 1 = exposure), as proposed by the WHO (WHO, 2011), was used to calculate participants’ total ACE scores (range = 0–13). In the present study, scores from the ACE obtained a Cronbach’s alpha coefficient of 0.848.

#### 2.3.3 Posttraumatic Stress Checklist for DSM-5

**Figure 1.**
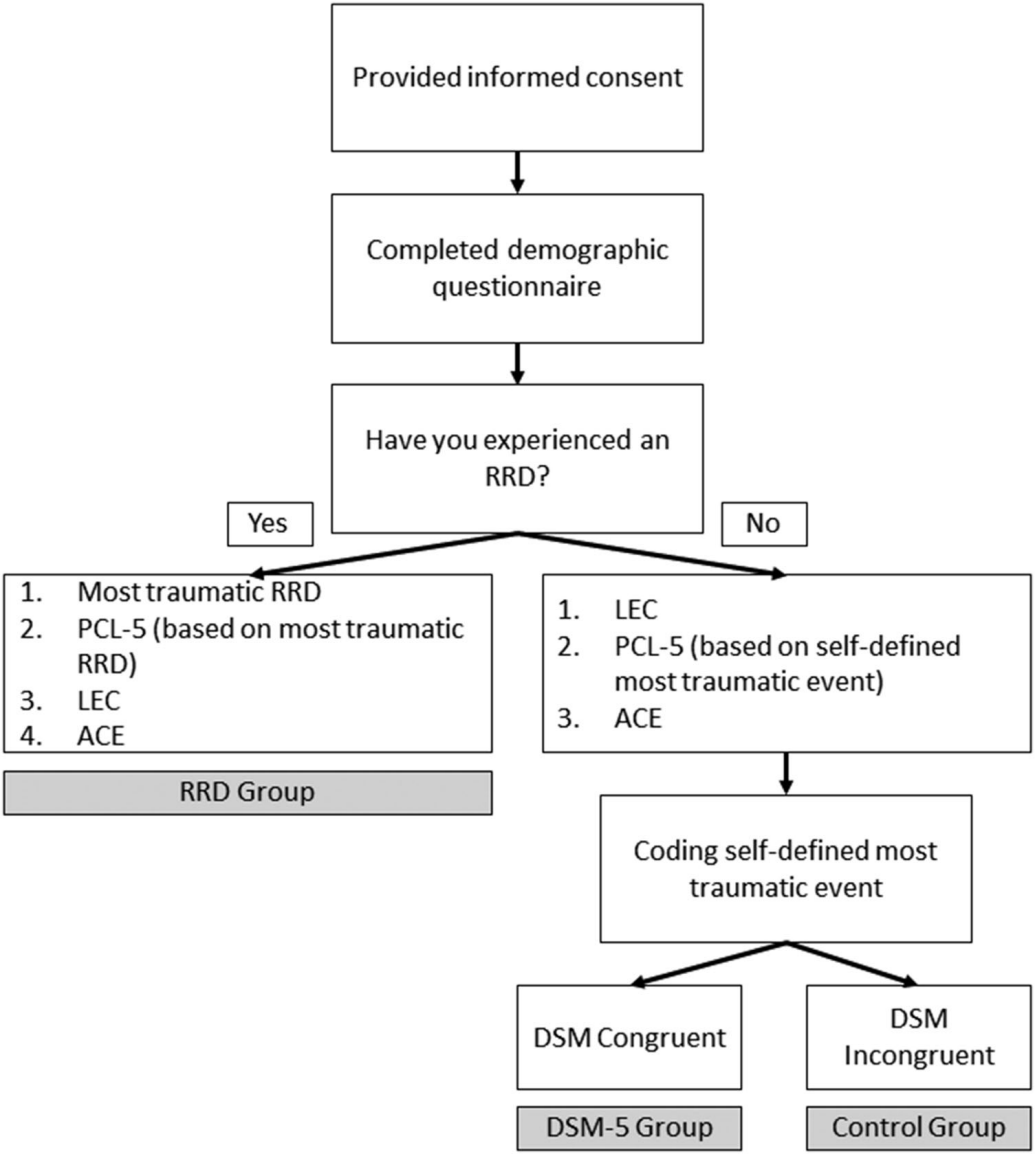
Group allocation procedure.

The PCL-5 is a self-report measure that assesses the 20 DSM-5 symptoms of PTSD and can be used to screen individuals for PTSD (Weathers et al., 2013). The PCL-5 consists of 20 items scored on a Likert-scale ranging from 1 = not at all, to 4 = extremely. A total symptom severity score (ranging from 0 to 80) can be obtained by summing the scores of all 20 items. The PCL-5 has demonstrated good reliability (*α *= 0.96) and convergent and discriminant validity (Bovin et al., 2016). Generally, a score of 31–33 is seen as indicating significant PTSS (National Center for PTSD 2012). Participants were asked to focus on a single specific event when completing the PCL-5 (see Figure 1). For the RRD group, responses were based on a self-defined most traumatic RRD, for the DSM-5 group on their most traumatic life event (identified on the LEC), and for the control group, on a self-defined most stressful event (non-criterion A event identified on the LEC). In the present study, scores from the PCL-5 obtained a Cronbach’s alpha coefficient of 0.946.

### 2.4 Case-case–control design and group allocation

We used a case-case–control design:

- RRD group: This group endorsed current PTSS in relation to a single specific RRD event.
- DSM-5 trauma group: This group endorsed PTSS in relation to a single specific DSM-5 congruent event for PTSD.
- Control group: This group endorsed PTSS in relation to another single stressful event (not an RRD and not a DSM-5 criterion A event).

Following the section on previous relationship information, participants were asked to indicate if they had experienced an RRD. Participants who answered ‘yes’ were diverted to a questionnaire asking about their most traumatic RRD experience, followed by the PCL-5, the LEC, and the ACE. Participants who indicated ‘no’ were diverted to a questionnaire containing the LEC, PCL-5, and the ACE, in that order.

Self-defined ‘*most traumatic’* events (see Life Events Checklist section) were then manually coded by the first author either as ‘DSM congruent’ or ‘DSM incongruent’ to differentiate the DSM-5 trauma and control groups, respectively. This process is depicted in Figure 1. The RRD group (*n *= 886), DSM-5 trauma group (*n *= 592), and control group (*n *= 544) were compared in terms of their socio-demographic information, attachment style, and trauma-related exposure. Additionally, we compared the time since their indexed (RRD, DSM-5 Criterion A, or other stressful event) trauma.

### 2.5 Analysis plan

Data were analysed using SPSS version 25 (IBM Corp, 2017) and Statistica version 13.5 (Dell, 2014). Based on a G*Power (Erdfelder et al., 2009) analysis the minimum sample required for an Analysis of Covariance (ANCOVA) with three groups and eight covariates is 400, indicating an adequate sample size.

Continuous variables (e.g. age) are expressed as means and standard deviations (SDs) while categorical variables (e.g. sex) are reported as frequency distributions. Chi-square tests (for categorical variables) and analyses of variance (ANOVA) (for continuous variables) were run to compare groups on variables such as age, time since exposure, sex, ethnicity, income, religious orientation, sexual orientation, current relationship status, and attachment style. When statistically significant differences were present, Games-Howell post hoc tests were run to establish group differences. Variables that differed statistically between the groups were deemed possible covariates, along with other pre-determined covariates, in further analyses.

#### 2.5.1 Determining variables associated with PTSS

The association of trauma-related exposure variables, attachment style, and socio-demographic factors (including sex, sexual orientation, current relationship status, and time since exposure) with PTSS scores were analysed using Pearson’s correlations (for continuous variables) and ANOVAs (for categorical variables) for each group separately, as well as for the total sample. Cohen’s effect sizes *d* (Cohen, 1988) were determined. An effect size of *d *= 0.2 is small, *d *= 0.5 is medium, and *d *= 0.8 is large. We also examined differences in the associations through slope analyses, using ANCOVA. Variables that were significantly associated with PTSS were considered as covariates for further analyses.

#### 2.5.2 Comparing PTSS levels following an RRD or a DSM-5 criterion A trauma event

To determine differences in PTSS scores following an RRD, a DSM criterion A trauma event, or another stressful event (control group), we compared the three groups on the PCL-5 using an ANCOVA with the significance level set at 0.05, while adjusting for possible covariates (as identified above). We then conducted a hierarchical regression analysis. In Step 1, all covariates were added to the model with the total PCL score as the outcome variable. In Step 2, the 3 groups were added and an *F* to remove test was conducted to determine whether there was a significant difference in the variance explained between Steps 1 and 2. The control group served as the reference group, while dummy variables were created for the DSM-Congruent and RRD Groups.

## 3. Results

As indicated in Table 2, the groups differed significantly on income (*p *= .02), sexual orientation (*p *< .01), current relationship status (*p *< .01), and attachment style (*p *< .01). Notably, the RRD Group had a greater proportion of anxious-ambivalent participants (22.5%) than the DSM-5 trauma group (15.7%); while the RRD Group had a lower proportion of secure (34.2%) and avoidant (43.3%) participants than the DSM-5 trauma group (secure = 37.5%; avoidant = 46.8%). There was a significant difference in time since event exposure (*p *< .01) between the groups.

**Table 2.** Between-group comparisons of socio-demographic characteristics, attachment style, trauma-related exposure, and time since index trauma.

	RRD Group (*n *= 886)	DSM-5 trauma group (*n *= 592)	Control group (*n *= 544)	*p*
Age Mean (years) SD (years)	20.52 1.86	20.59 1.97	20.57 1.87	.83
Time since exposure Mean (months) SD (months)	10.39 12.41	49.16 49.65	34.57 44.77	<.01
LEC Total Mean (SD) Mode	5.08(3.67) 2	5.61(3.49) 3	3.31(3.04) 0	<.01
ACE Total Mean (SD) Mode	2.96(2.55) 0	2.85(2.43) 1	2.08(20.4) 1	<.01
** **	***n*(%)**	***n*(%)**	***n*(%)**	***p***
Sex Male Female Intersexual	265(29.9) 621(70.1) -	152(25.7) 439(74.2) 1(0.2)	164(30.1) 378(69.5) 2(0.4)	.14
Ethnicity African/black Mixed race/coloured Indian White Other Prefer not to say	127(14.3) 175(19.8) 25(2.8) 527(59.5) 7(0.8) 24(2.7)	102(17.2) 98(16.6) 19(3.2) 356(60.1) 5(0.8) 12(2.0)	81(14.9) 82(15.1) 18(3.3) 343(63.1) 10(1.8) 8(1.5)	.17
Monthly income of main breadwinner Do not know R 0–R 11 352 (0 USD-750.70 USD) R 11 353–R 30 164 (750.76 USD–1 994.72 USD) R 30 165–R 68 528 and above (1 994.79 USD–4 531.70 USD and above)	182(20.5) 132(14.9) 203(22.9) 365(41.2)	114(19.3) 98(16.6) 140(23.6) 236(39.9)	139(25.6) 79(14.5) 143(26.3) 179(32.9)	.02
Religious orientation I am not religious Christian Other	242(27.3) 559(63.1) 59(6.7)	161(27.2) 375(63.3) 33(5.6)	138(25.4) 364(66.9) 24(4.4)	.36
Sexual orientation Heterosexual Other	684(77.2) 198(22.3)	470(79.4) 119(20.1)	462(84.9) 81(14.9)	<.01
Current relationship status Single Not single	624(70.4) 262(29.6)	255(43.1) 337(56.9)	266(48.9) 278(51.1)	<.01
Attachment Style Secure Avoidant Anxious-ambivalent	303(34.2) 384(43.3) 199(22.5)	222(37.5) 277(46.8) 93(15.7)	234(43.0) 246(45.2) 64(11.8)	<.01

Note: SD = Standard Deviation; USD = Unites States Dollar; United States Dollar equivalents were calculated on the exchange rate as on 1 March 2021. ACE = Adverse Childhood Experiences questionnaire; LEC = Life Events Checklist.

Games-Howell (LSD) post hoc analysis indicated a significant difference in total LEC mean scores between RRD and DSM-5 trauma groups (*p *= .01; Cohen’s *d *= 0.15), between RRD and control groups (*p *< .01; Cohen’s *d *= 0.51), and between DSM-5 trauma and control groups (*p *< .01; Cohen’s *d *= 0.70). Similarly, LSD tests indicated a significant difference in ACE mean scores between RRD and control groups (*p *< .01; Cohen’s *d *= 0.37), and between DSM-5 trauma and control groups (*p *< .01; Cohen’s *d *= 0.34). However, there was no significant difference in mean ACE scores between RRD and DSM-5 trauma groups (*p *= .70; Cohen’s *d *= 0.04).

### 3.1 Variables associated with PTSS

In the full sample, the mean (35.75; SD = 20.46, mode = 0) total PCL-5 score was above the cut-off score for probable PTSD (National Center for PTSD 2012). Time since exposure was negatively correlated with total PCL-5 scores (*r *= −0.078; *p *< .01). While there was a significant difference in the association between time since exposure and total PCL-5 score for the groups (*p *= .02), the association was weak and statistical significance may have been reached due to the large sample.

Further, total LEC scores were positively correlated with total PCL scores (*r *= 0.298; *p *< .01). Post-hoc (LSD) analysis indicated a significant difference between the RRB (*r *= 0.184) and DSM-5 trauma (*r *= 0.298) groups (*p *= .02), and between the RRB and control (*r *= 0.368) groups (*p *< .01), but not between the DSM-5 trauma and control groups (*p *= .19).

Similarly, total ACE scores were positively correlated with total PCL-5 scores (*r *= 0.368; *p *< .01). However, LSD tests did not indicate any significant difference in this association between the RRB (*r *= 0.298) and DSM-5 trauma (*r *= 0.382) groups (*p *= .08), between the RRB and control (*r *= 0.368) groups (*p *= .08), or between the DSM-5 trauma and control groups (*p *= .96).

As indicated in Table 3, other variables significantly associated with total PCL-5 scores included sex, monthly income, sexual orientation, and attachment style. This supports our hypothesis that mean trauma exposure, anxious-ambivalent attachment style, being female, and having a minority sexual orientation would positively correlate with PTSS levels. However, contrary to our hypothesis, relationship status was not significantly associated with PTSS scores.

**Table 3.** Factors associated with total PCL-5 scores and intergroup differences.

Variable	RRB group				DSM-5 trauma group				Control group				Total group				Intergroup difference
	*r*		*p*		*r*		*p*		*R*		*p*		*r*		*p*		
Age	0.025		.449		0.037		.370		0.072		.092		0.037		.100		0.63
Time since exposure (months)	0.066		.052		−0.058		.157		0.071		.096		−0.078		<.01		0.02
LEC	0.184		<.01		0.298		<.01		0.368		<.01		0.298		<.01		<0.01
ACE	0.298		.01		0.382		<.01		0.384		<.01		0.368		<.01		<0.01
Variable	Total PCL score		F (*p*)	η2	Total PCL score		F (*p*)	η2	Total PCL score		F (*p*)	η2	Total PCL score		F (*p*)	η2	Intergroup difference
	Means	SE			Mean	SE			Means	SE			Means	SE			
Sex Male Female Intersexual	37.78 43.65 -	1.11 0.74 -	18.88 (<.01)	0.02*	26.69 37.55 25.00	1.57 0.93 -	17.786 (<.01)	0.06**	21.98 28.92 39.00	1.53 1.06 8.00	7.04 (.001)	0.03*	30.42 37.91 34.33	0.83 0.53 6.27	28.49 (<.01)	0.03*	0.09
Monthly income of main breadwinner Do not know R0 – R11352 R11353–R30164 R30165–R68528 and above	42.71 44.11 42.58 10.46	1.35 1.72 1.33 0.95	1.56 (.198)	0.005	34.54 39.48 35.08 32.50	1.91 2.03 1.74 1.24	2.876 (.036)	0.02*	23.27 26.35 32.35 24.99	1.65 2.42 1.65 1.51	5.57 (.001)	0.03*	34.35 38.10 37.41 34.20	1.00 1.22 0.91 0.71	4.091 (.007)	0.006	0.03
Religious orientation I am not religious Christian Other	43.64 40.94 445.24	1.13 0.81 2.29	2.708 (.067)	0.006	36.31 33.31 41.42	1.56 1.04 3.47	3.290 (.038)	0.01*	31.34 24.94 29.25	1.84 1.06 3.70	5.07 (.007)	0.02*	38.32 34.25 40.84	0.86 0.58 1.79	11.311 (<.01)	0.01*	0.48
Sexual orientation Heterosexual Other	40.72 45.79	0.70 1.34	11.586 (.001)	0.01*	32.99 41.77	0.91 1.80	18.948 (<.01)	0.03*	26.00 32.11	0.93 2.54	6.182 (.013)	0.01*	34.26 41.81	0.50 1.03	44.37 (<.01)	0.02*	0.34
Current relationship status Single Not single	40.80 44.50	0.74 1.15	7.354 (.007)	0.008	33.44 35.72	1.26 1.08	1.901 (.168)	0.003	26.71 27.01	1.27 1.23	0.030 (.863)	0.000	35.89 35.58	0.60 0.70	0.111 (.739)	0.000	0.31
Attachment style Secure Avoidant Anxious-ambivalent	35.35 43.25 49.24	1.11 0.91 1.08	38.388 (<.01)	0.08**	28.64 37.97 39.68	1.32 1.15 2.02	17.805 (<.01)	0.06**	21.15 29.13 39.00	1.21 1.28 2.82	23.579 (<.01)	0.08**	29.01 37.81 44.90	0.73 0.66 0.98	88.40 (<.01)	0.08**	0.22

Note: *small effect (>0.01); **medium effect (> 0.06); ***large effect (> 0.14); LEC = Life Events Checklist; ACE = Adverse Childhood Experiences questionnaire.

### 3.2 PTSS and probable PTSD following an RRD or a DSM-5 criterion A trauma event

As indicated in Table 4, there was a significant difference in mean total PCL-5 scores among the groups, with *F*(2, 2019) = 101.09, *p *< .01. Specifically, the RRD group had the highest mean total PCL-5 score (LS mean = 41.89), while the control group had the lowest mean total PCL-5 score (LS mean = 26.86). Further, the RRD group scored the highest on all the symptom clusters, with the exception of Cluster C (avoidance). All post hoc analyses (Games-Howel and LSD) were significant between all the groups (*p *< .01).

**Table 4.** Between-group comparison of posttraumatic stress symptoms and symptom clusters, as measured by the PCL.

	RRD group (*n *= 886)	DSM-5 trauma group (*n *= 592)	Control group (*n *= 544)	*p*
PCL-5 Total LS Mean SE	41.89 0.66	34.74 0.80	26.86 0.84	<.01
Cluster B Mean SD Range	10.64 5.461 0–20	9.22 5.503 0–20	6.93 5.697 0–20	<.01
Cluster C Mean SD Range	2.13 2.397 0–8	4.31 2.740 0–8	3.47 2.850 0–8	<.01
Cluster D Mean SD Range	14.87 7.398 0–28	11.47 7.910 0–28	9.05 7.987 0–28	<.01
Cluster E Mean SD Range	11.42 6.226 0–24	9.74 6.403 0–24	7.42 6.319 0–24	<.01
** **	***n* (%)**	***n* (%)**	***n* (%)**	***p***
Probable PTSD based on PCL-5 scores No Yes	265(29.9) 621(70.1)	264(44.6) 328(55.4)	326(59.9) 218(40.1)	<.01

Note: Cluster B = Re-experiencing; Cluster C = Avoidance; Cluster D = Negative alterations in cognition and mood; Cluster E = Hyper-arousal; LS = Least Squares Mean; PCL = Posttraumatic stress Check List; PTSD = posttraumatic stress disorder based on cut-off score of 33; SE = Standard Error.

Further, for both the RRD and DSM-5 trauma groups, the mean total PCL-5 score was above the clinical cut-off of 33 for probable PTSD (National Center for PTSD, 2012). Within the RRD group, 72.9% scored above the clinical cut-off score of 33 and above; 55.4% in the DSM-5 Trauma group; and 40.1% in the control group. As indicated in Table 4, there was a significant between-group difference in the number of participants meeting symptom criteria for PTSD, with *F *= 72, 35, df = 2, *p *< .01. Post hoc (Games-Howell) tests indicated a significant difference between all the groups (*p *< .01).

For the ANCOVA, total PCL-5 scores were entered as the dependant variable, while group status (i.e. RRD, DSM-5, and Control group) was entered as the independent variable. Total PCL-5 scores remained significantly different after controlling for mean total ACE scores, mean LEC scores, months since exposure, sex, monthly income, sexual orientation, current relationship status, and attachment style *F *= 64.702, df = 2, *p *< .01 (see Table 5).

**Table 5.** ANOCVA results testing the effect of group status on PCL-5 scores.

	*df*	*F* value	*p*
**(Intercept)**	1	726.57	<.01
Time since exposure (months)	1	0.26	.61
LEC Total	1	67.02	<.01
ACE Total	1	113.09	<.01
Sex	1	37.24	<.01
Monthly income	3	1.46	.22
Sexual orientation	1	7.35	.01
Current relationship status	1	6.65	.01
Attachment style	2	38.99	<.01
**Group**	**2**	**64**.**70**	**<**.**01**

Note: Grou*p* = RRD Group, DSM-5 Trauma Group, or Control Group.

Table 6 presents the stepwise hierarchical regression analysis results. As can be seen from Step 1, the covariates explained 24% of the variance in total PCL-5 scores, which increased to 28% in Step 2 when the group variable was added. The *F* to remove test indicated that the difference in variance explained was significant with *R*^2 ^= 0.29, Δ*R*^2 ^= 0.05, *p *< .01. Therefore, group status explained significantly more of the variance in total PCL-5 scores.

**Table 6.** Stepwise hierarchical regression analysis indicating the effect of the group variable.

Step 1	b*	SE of b*	*B*	SE of *b*	*t*(1953)	*p*-value
Intercept			26.44	1.62	16.33	<.01
Time since exposure (months)	−0.09	0.02	−0.04	0.01	−4.40	<.01
LEC Total	0.20	0.05	1.15	0.12	9.53	<.01
ACE Total	0.26	0.02	2.17	0.19	11.49	<.01
Sex (male)	−0.12	0.02	/5.34	0.91	−5.89	<.01
Monthly income (R 0–R 11 352)	−0.05	0.02	−2.93	1.40	−2.10	.04
Monthly income (R 11 353–R 30 164)	0.00	0.03	0.19	1.19	0.16	.88
Monthly income (R 30 165–R 38 528 and above)	0.01	0.03	0.31	1.08	0.29	.77
Sexual orientation (heterosexual)	−0.06	0.02	−3.14	1.04	−3.01	<.01
Relationship status (single)	−0.01	0.02	−0.34	0.83	−0.42	.68
Attachment style (Avoidant)	0.11	0.02	4.57	0.93	4.92	<.01
Attachment style (Anxious-Ambivalent)	0.20	0.02	10.94	1.20	9.13	<.01
***R *= 0.491; *R*^2 ^= 0.241; Adjusted *R*^2 ^= 0.236; *F*(11, 1953) = 56.287; SE of Estimate = 17.795; *p *< .01**						
Step 2	b*	SE of b*	*b*	SE of *b*	*t*(1951)	*p*-value
Intercept			21.51	1.65	13.07	<.01
Time since exposure (months)	−0.01	0.02	−0.01	0.01	−0.51	.61
LEC Total	0.17	0.02	0.98	0.12	8.19	<.01
ACE Total	0.23	0.02	1.96	0.18	10.63	<.01
Sex (male)	−0.12	0.02	−5.37	0.88	−6.10	<.01
Monthly income (R 0–R 11 352)	−0.01	0.02	−2.32	1.35	−1.71	.09
Monthly income (R11 353–R30 164)	0.01	0.02	0.25	1.16	0.21	.83
Monthly income (R 30 165–R 38 528 and above)	−0.00	0.03	−0.18	1.05	−0.17	.87
Sexual orientation (heterosexual)	−0.05	0.02	−2.74	1.01	−2.71	.01
Relationship status (single)	−0.05	0.02	−2.12	0.82	−2.58	.01
Attachment style (Avoidant)	0.12	0.02	4.83	0.90	5.37	<.01
Attachment style (Anxious-Ambivalent)	0.19	0.02	10.10	1.16	8.68	<.01
Group (RRB)	0.28	0.03	11.41	1.04	11.02	<.01
Group (DSM-5 Trauma)	0.08	0.02	3.55	1.08	3.28	<.01
***R *= 0.537; *R*^2 ^= 0.288; Adjusted *R*^2 ^= 0.283; *F*(13, 951) = 60.688; SE of Estimate = 17.241; *p *< .01**						

## 4. Discussion and clinical implications

To our knowledge, this is the first study indicating, that RRDs are significantly associated with significantly more PTSS than DSM-5 criterion A traumas among EAS, even after controlling for variables significantly associated with PTSS. This finding supports the ongoing debate on the theoretical and practical validity of Criterion A. Furthermore, in terms of probable PTSD (using a PCL-5 cut-off score of 33 and above), an RRD was more likely to be associated with probable PTSD compared to a DSM-5 criterion A trauma event. RRD participants also reported higher re-experiencing symptoms, negative alterations in cognition and mood, and hyper-arousal than the DSM-5 Trauma group. Our findings are consistent with previous research indicating that RRDs are often associated with severe distress (Owenz & Fowers, 2019) and PTSS (Chung et al., 2002; Chung, Farmer, et al., 2002; Chung et al., 2003; Studley & Chung, 2015). This underscores the impact that RRDs may have despite often being ‘dismissed or trivialised as a rite of passage’ (Belu et al., 2016). Indeed, events that are not culturally or objectively acknowledged as a trauma – such as RRDs among EAS – may confer more risk on account of a perceived lack of social support and acceptance (Bui et al., 2012; Ladois-Do Pilar Rei et al., 2012; Lansing et al., 2017). If an RRD, as a non-criterion A event, is discounted by student counselling services and traumatic responses are deemed to be ‘overblown’ (p. 41), this may impede help-seeking behaviour and serve as a barrier to treatment (Lansing et al., 2017) for EAS who may benefit from treatment. Validating the experience of RRDs in EAS may enhance positive outcomes (Wrape et al., 2016). Further, for emerging adults who present with PTSS following an RRD, treatment modalities for PTSD that have proven efficacy may be appropriate. For example, Prolonged Exposure Therapy may assist during a time where complete avoidance of an ex-partner (e.g. on social media or as a classmate) may be difficult.

Our study also provides evidence that trauma-related exposure, attachment style, and socio-demographic factors (including sex, sexual orientation, time since trauma exposure, and income) were associated with PTSS levels in EAS. Since EAS already face academic, family, personal, and financial stress (Hurst et al., 2012) it is important to identify additional cumulative stressors that may increase the vulnerability of developing PTSD following additional stressors (van der Watt et al., 2023).

As expected, trauma-related exposure (including lifetime traumatic events and adverse childhood events) was significantly and positively associated with PTSS. Thus, the more trauma-related exposure participants had, the higher their PTSS scores; consistent with previous studies (American Psychiatric Association, 2013; Lansing et al., 2017, 2016; Neria et al., 2008). Student counselling services should provide targeted interventions for at-risk EAS, such as those with prior traumatic events, multiple traumatic events (especially interpersonal traumas), and adverse childhood events exposure, to mitigate the effects of additional everyday stressors.

Attachment style was significantly associated with PTSS, with an insecure attachment style associated with increased PCL-5 scores. This finding is in accordance with the literature (O’Connor & Elklit, 2008; Woodhouse et al., 2015) showing that an insecure (avoidant or anxious-ambivalent) attachment style is associated with higher levels of PTSS. It should be noted, however, that this relationship is bi-directional with the possibility that the measured attachment style may be a consequence of the RRD. Nonetheless, a participant with a secure attachment style may exhibit less PTSS following a traumatic event, an RRD, or any other stressful event. This may be linked to better emotion regulation in securely attached individuals (Mikulincer & Shaver, 2007). Specifically, interventions for PTSS prevention following RRDs, traumatic, and stressful events should focus on insecurely attached EAS. Student development and counselling services could make an important psychoeducational contribution by making students aware of and provide information about the influence of attachment style and associated emotional regulation deficits. This may encourage students to self-identify developmental needs in this area and preventatively seek out counselling services.

As hypothesised, sex was significantly associated with PTSS, with female sex being associated with higher levels of PTSS. However, the effect size was small (§ = 0.03). Similarly, a significant association of sex (but with small effect size) and breakup distress as measured by the Breakup Distress Scale was found. Therefore, while our findings support a body of evidence that women are more likely to develop PTSS following traumatic events (Christiansen & Berke, 2020; Christiansen & Elklit, 2011; Shansky, 2015), the emotional response of male students following an RRD must not be overlooked. This finding is noteworthy considering sex differences in help-seeking for mental illness (Juvrud & Rennels, 2017), with men being less likely to seek help from mental health professionals (Mackenzie et al., 2006). Thus, it is important for student counselling services to encourage help-seeking behaviour among male EAS who have experienced an RRD, traumatic, or stressful event.

Sexual orientation was significantly associated with PTSS. In accordance with prior research (Lannutti & Cameron, 2002; Roberts et al., 2010) minority sexual orientation (e.g. LGBTQI+) was associated with higher levels of PTSS compared with a heterosexual orientation. This highlights the importance for student counselling services to provide targeted interventions in minority sexual orientation EAS following RRDs, traumatic, and stressful events.

Contrary to our hypothesis, current relationship status was not significantly associated with PTSS. This is in contrast with previous literature indicating that being in a relationship may serve as a protective factor against stress (Israel-Cohen & Kaplan, 2016) and in the development of PTSS (Weisenhorn et al., 2017). From an attachment theory perspective, this finding is surprising given that an attachment figure provides safety and security (Bowlby, 1969; Fraley & Shaver, 2000; Hazan & Shaver, 1987). However, this association may have been influenced by the binary coding of single versus not-single. There are different ways to define what counts as ‘being single’ or ‘being in a relationship’ (Del Russo, 2018; Rus & Tiemensma, 2017; van der Watt, 2015), especially in this modern era and in popular media. For example, one website listed 11 different types of relationship statuses (Coetzee, n.d.), while another also listed 11 different types (Tepfenhart, n.d.) that did not necessarily overlap. Thus, a person may be ‘seeing each other’ or a have a ‘backup’ – which both involve spending time together, often including having sex – but still be defined as being single (Coetzee, n.d.; Tepfenhart, n.d.). Therefore, some of our study participants may have benefited from a traditional relationship arrangement (e.g. companionship, physical intimacy) while still reporting being single.

Time since exposure was negatively correlated with PTSS levels with more time since exposure (albeit trauma, an RRD, or any other stressful event) associated with lower PTSS levels. This supports research indicating that time since trauma exposure may have a moderating role in the development of PTSS following a traumatic (Weems & Carrion, 2007) or stressful event (Field et al., 2009). Interestingly, in the same sample, time since the RRD was not significantly associated with breakup distress (Van der Watt et al., 2022) as measured by the Breakup Distress Scale (Field et al., 2009). Further longitudinal research is needed to better understand the timing of trauma- and distress-related symptoms following an RRD.

Lastly, our findings indicate that income level is significantly associated with PTSS. Specifically, a higher income was associated with lower PTSS scores. This is consistent with research indicating that social ecological factors (DiGangi et al., 2013) such as income, are associated with PTSS. Specifically, lower income associated with poverty (Koenen et al., 2007) may be a risk factor for PTSD. This highlights the importance of providing affordable mental health care to students from lower income families.

### 4.1 Limitations and future recommendations

This study had limitations. First, the sample, albeit of large size, was limited to EAS from a single university and not necessarily representative of South African students at other universities. The sample also had an over-representation of women. Future research should include a more diverse and representative sample.

Second, electronic surveys have been criticised for having several limitations including a lower response rate and response bias (Bethell et al., 2004; Vrijheid et al., 2009). However, research also indicates that response bias may not be appreciably different between paper and online surveys (Menachemi, 2011; Sax et al., 2008). It is also possible that the response rate was impacted by both university examinations and university holidays spanning the period of data collection (1 August 2019 and 20 March 2020). However, in this study the response rate was satisfactory, and the resulting sample was representative of the university population (Stellenbosch University, 2018). Indeed, the present study’s response rate was higher (7.9%) than a previous online study regarding romantic loneliness (6.3%) conducted among the same population (Lesch et al., 2016). Further, using G*Power (Erdfelder et al., 2009) the minimum sample required for an ANCOVA with three groups and eight covariates is 400, clearly indicating an adequately powered study. Nonetheless, we encourage future research to include paper-and-pencil surveys to increase response rates and generate a more diverse sample (e.g. including participants who may not have sufficient internet access, such as those with a lower income). Additionally, while significant, these findings need to be replicated in a community sample who are less educated.

Third, we acknowledge that in terms of RRDs, various factors related to the relationship and dissolution may have influenced PTSS severity. Indeed, in the present sample total prior RRDs, perceived closeness, initiator status, wanting the relationship to end, expecting the relationship to end, feeling betrayed, wanting to renew the relationship, and various other reasons for the dissolution (including trust, general differences, external reasons, and *just because*) were significantly associated with breakup distress (van der Watt et al., 2022) as measured by the Breakup Distress Scale (Field et al., 2009). However, since these factors were unique to the RRD group, and not the focus of the present article, these findings are reported elsewhere.

Fourth, as mentioned earlier, due to the extensive nature of the main study’s online survey, we used a single self-report measure for attachment style to mitigate participant fatigue. While this is a valid and widely used measure, a more comprehensive assessment of attachment (e.g. one relating to the specific former romantic relationship in question) may enhance our understanding of the role of attachment style in PTSS severity.

Fifth, it should be noted that PCL-5 scores in the sample were generally high. An alternative method of determination of a symptom level diagnosis of PTSD, for example applying the DSM-5 criteria of at least one intrusion symptom, one avoidance symptom, two negative alterations in cognitions and mood symptoms, and two arousal symptoms may have yielded a lower rate. Indeed, a meta-analysis found a stronger relationship between PTSS and traumatic events than DSM-5 incongruent traumas (‘stressors’), however, the effect size was small (0.18) and there was an overlapping distribution of PTSS in the ‘trauma’ and ‘stressor’ groups (Larsen & Pacella, 2016). The authors highlight the importance of considering moderating factors for PTSD that may further serve to elucidate differences between traumas and stressors.

Lastly, while we attempted to control for factors which may influence PTSS levels (including trauma-related exposure, attachment style, and socio-demographic factors) when determining the difference in PTSS levels between the three groups, other factors (e.g. specific religious orientations and denominations) may have contributed to the significant difference in PTSS levels. In the present study, religion was not included as a covariate since between-group differences were not significant. However, religious orientation and denomination (e.g. Catholicism, Protestant, and Evangelism) are more nuanced and may need to be considered. Similarly, more nuanced relationship categories need to be considered in future research. We recommend that future studies consider other contributory factors for PTSS following an RRD or other traumatic event.

## 5. Conclusion

Non-marital RRDs frequently occur among emerging adult students and are associated with significant PTSS. Indeed, in the present study, EAS’ PTSS based on a non-marital RRD was significantly higher than that of students exposed to a DSM-5 Criterion A traumatic event. Factors associated with PTSS following an RRD or DSM-5 Criterion A traumatic event include attachment style, sex, sexual orientation, and monthly income level. Student counselling services should encourage help-seeking behaviour especially among male EAS. Targeted interventions for PTSS prevention following RRDs, traumatic, and stressful events among minority sexual orientation, single, and lower-income students should be considered. Our data show that RRDs are traumatic events that are associated with significant PTSS and may be a valid criterion for a PTSD diagnosis. This brings into question the validity of Criterion A as gatekeeper for a PTSD diagnosis.

## Data Availability

The data used are available from the first author upon reasonable request.

## Notes

1 It should be noted that in terms of RRDs specifically, various factors related to the relationship and dissolution of the relationship may influence levels of distress or PTSS. This includes who initiated the breakup e.g., (Akbari et al., 2022), whether the breakup was expected or not e.g., (Attridge et al., 1995), reasons for the breakup, the duration e.g., (Dailey et al., 2020) and perceived closeness of the relationship prior to dissolution, the breakup strategy, and feeling betrayed by the ex-partner e.g., (Koessler et al., 2019). However, since these influences are unique to those exposed to an RRD, they were not considered in the present analysis but have been reported separately.

2 Ideally, we would have used a more comprehensive assessment of attachment (e.g., Experience in Close Relationships scale). However, due to the already extensive nature of the online survey (including the Inventory of Complicated Grief and the Breakup Distress scale not reported on in this article), we opted to use Hazan and Shaver’s (1987) widely used single self-report measure for romantic attachment style.
